# The Transformative Impact of the African Cohort Study (AFRICOS) Toward Reaching HIV 95-95-95 Goals in Sub-Saharan Africa

**DOI:** 10.4269/ajtmh.24-0249

**Published:** 2024-10-29

**Authors:** Neha Shah, Trevor A. Crowell, Jaclyn Hern, Victor Anyebe, Emmanuel Bahemana, Hannah Kibuuka, Valentine Singoei, Jonah Maswai, Ajay Parikh, Emma R. Duff, Joseph Sean Cavanaugh, Matthew L. Romo, Julie A. Ake, Michael J. A. Reid, AMB John N. Nkengasong, the AFRICOS Study Group

**Affiliations:** ^1^ U.S. Military HIV Research Program, Center of Infectious Disease Research, Walter Reed Army Institute of Research, Silver Spring, Maryland;; ^2^ Henry M. Jackson Foundation for the Advancement of Military Medicine, Inc., Bethesda, Maryland;; ^3^ U.S. Military HIV Research Program, Walter Reed Army Institute of Research Africa, Abuja, Nigeria;; ^4^ Henry Jackson Foundation Medical Research International, Abuja, Nigeria;; ^5^ U.S. Military HIV Research Program, Walter Reed Army Institute of Research Africa, Mbeya, Tanzania;; ^6^ HJF Medical Research International, Mbeya, Tanzania;; ^7^ Makerere University Walter Reed Project, Kampala, Uganda;; ^8^ U.S. Military HIV Research Program, Walter Reed Army Institute of Research, Walter Reed Army Institute of Research Africa, Kisumu, Kenya;; ^9^ HJF Medical Research International, Kisumu, Kenya;; ^10^ U.S. Military HIV Research Program, Walter Reed Army Institute of Research, Walter Reed Army Institute of Research Africa, Kericho, Kenya;; ^11^ HJF Medical Research International, Kericho, Kenya;; ^12^ Bureau of Global Health Security and Diplomacy, President’s Emergency Plan for AIDS Relief, Washington, District of Columbia;; ^13^ Institute of Global Health Sciences, University of California at San Francisco, San Francisco, California

## Abstract

Over the last 20 years, the U.S. President’s Emergency Plan for AIDS Relief (PEPFAR) has rapidly expanded and made remarkable progress toward the UNAIDS 95-95-95 targets to end the HIV epidemic. Nevertheless, HIV continues to pose a significant health challenge globally, with a particular impact on the African continent. Funded by PEPFAR, the African Cohort Study (AFRICOS) has served as a monitoring and evaluation tool for PEPFAR to help guide HIV policy and PEPFAR programming for the last 10 years since its inception and offers a compelling example of how PEPFAR’s investment in science continues to reap dividends. This paper details and critically reviews the transformative research AFRICOS has had on helping to end the HIV epidemic as a public health threat by 2030.

## INTRODUCTION

When the HIV epidemic began, there were profound effects on countries’ social, economic, and healthcare landscapes. Unfortunately, Africa bore a disproportionate burden of the global HIV/AIDS pandemic, with nearly two-thirds of all people living with HIV residing on the continent. However, significant progress has been made in controlling the epidemic. According to the Joint UN Program on HIV/AIDS (UNAIDS), the annual number of new infections decreased from 1.6 million in 2010 to approximately 1.3 million in 2022. In addition, the number of AIDS-related deaths in Africa declined from 1.3 million in 2010 to approximately 630,000 in 2022.^1^

The U.S. President’s Emergency Plan for AIDS Relief (PEPFAR) was established in 2003 in response to the escalating HIV crisis. Recognizing the need for a comprehensive and coordinated effort, PEPFAR works alongside partner governments, civil society organizations, private sector organizations, and external partners to control the HIV/AIDS pandemic. This collaborative effort has led to lifesaving antiretroviral therapy (ART) being available and provided to 20.5 million people living with HIV (PLWH).^2^ In addition to ART, prevention efforts have intensified, with widespread educational campaigns, condom distribution, and increased awareness leading to a decline in new infections. However, challenges such as ensuring that the needs of the younger population living with HIV are met, ongoing stigma, and diagnosis and treatment of comorbidities persist.

Africa’s HIV landscape is complex, marked by diverse cultures, socioeconomic challenges, and variations in healthcare infrastructure. Understanding the nuances of the epidemic within this context is paramount for designing effective, culturally sensitive interventions. The African Cohort study (AFRICOS) was established in 2013 as a 15-year observational study to help guide PEPFAR programming and policy. Funded by PEPFAR, AFRICOS is a longitudinal observational cohort study of PLWH and a smaller group of people living without HIV (PLWoH) and runs in four countries within sub-Saharan Africa: Nigeria, Kenya, Tanzania, and Uganda. The African Cohort study is uniquely positioned to evaluate the longitudinal impact of clinical practices, biological factors, and socio-behavioral issues on disease progression and HIV infection in an African context. The robust data collected in the cohort allow for investigation of HIV disease progression and associations with a multitude of health outcomes to identify valuable insights that are described in later sections; further details of the cohort are described in prior publications and are available online at https://www.hivresearch.org/african-cohort-study-africos.^3^^,^^4^

The African Cohort study has provided valuable insights into the natural course of diseases, the effectiveness of interventions, and the identification of potential risk factors. The goal of this paper is to describe the contributions of AFRICOS to the continual effort to fight against HIV/AIDS in Africa and to reach the UNAIDS’ goals of ending the epidemic by 2030. As a summary of the breadth of scientific insights generated over the last decade, the paper also provides examples of how PEPFAR’s sustained investment in science continues to inform HIV programming decisions in Africa and beyond. Going forward, as countries progress toward the 95-95-95 targets, the importance of following the science could not be more relevant/pertinent. In the following sections, key results across the major domains/topic areas (i.e., mental health and cognition, clinical profile, and adherence) and their contribution to research are discussed, as well as how AFRICOS is looking to sustain the gains in the future.

## RESEARCH CONTRIBUTIONS

Over the past decade, the AFRICOS Study Group’s findings have contributed to our understanding of HIV epidemiology, clinical outcomes, and sociodemographic factors in the African context. These papers have informed how PEPFAR has shifted programmatic priorities with the evolving phases of the epidemic. Outlined below and in Table 1 are the key insights that have shaped PEPFAR’s response over the last 10 years.

1. *Clinical Profiles and Treatment Outcomes:* AFRICOS has examined the clinical characteristics of PLWH, including CD4 cell counts, viral loads, and treatment adherence. These studies have contributed to our understanding of disease progression, the efficacy of ARTs, and the factors influencing treatment outcomes.

The findings from papers looking at viral suppression indicate that the use of second-line ART, low CD4 count, and missing any day of ART are important predictors of virologic failure.^5^ These results were further confirmed when we applied machine learning methods to better characterize participants at risk of viral failure.^6^ From a programmatic standpoint, we evaluated the role of low-level viremia on subsequent virologic failure. Any viral load test results >200 copies/mL were associated with an increased risk of subsequent virologic failure.^7^^,^^8^

**Table 1:** Summary of AFRICOS studies, objectives, and key findings

Title	Reference	Objective	Primary Findings	Pub Year
Clinical Profiles and Treatment Outcomes				
Persistent Low-Level Viremia Predicts Subsequent Virologic Failure: Is It Time to Change the Third 90?	Esber et al.^7^	Determine If Persistent Low-Level Viremia (pLLV) Was Associated with Risk of Subsequent Virologic	Participants with pLLV ≥200 copies/mL were at increased risk of subsequent virologic failure.	2019
HIV Virologic Failure and Its Predictors among HIV-Infected Adults on Antiretroviral Therapy in the African Cohort Study	Kiweewa et al.^5^	Determine Factors Associated with Virologic Failure	The odds of virologic failure were associated with being on second-line ARTs, low CD4 count, and missing ART doses.	2019
Impact of Age on CD4 Recovery and Viral Suppression Over Time among Adults Living with HIV Who Initiated Antiretroviral Therapy in the African Cohort Study	Bahemana et al.^13^	Characterize CD4 Recovery and Viral Suppression among Adults Aged <50 and ≥50 Years Living with HIV Who Initiated ART	Examining CD4 recovery and viral suppression showed there were no significant differences by age group over time.	2020
Predictors of All-Cause Mortality among People with Human Immunodeficiency Virus (HIV) in a Prospective Cohort Study in East Africa and Nigeria	Kibuuka et al.^4^	To Describe All-Cause Mortality and Its Predictors in People Living with HIV (PLWH)	Most common causes of death were infection (29.7%) and malignancy (28.1%). Predictors of all-cause mortality were CD4, viral load, hemoglobin level, and low BMI.	2022
Brief Report: Virologic Impact of the Dolutegravir Transition: Prospective Results from the Multinational African Cohort Study	Esber et al.^8^	Described the Virologic Consequences of Switching from Non–Dolutegravir-Based Therapy to Tenofovir/Lamivudine/Dolutegravir (TLD) Regimens	Not switching to TLD had significantly higher rates of being virally unsuppressed.	2022
Achieving the Third 95 in Sub-Saharan Africa: Application of Machine Learning Approaches to Predict Viral Failure	Esber et al.^6^	To Compare the Performance of Two Supervised Learning Algorithms to Predict Viral Failure	CD4, ART regimen, age, self-reported adherence, and duration on ART were important factors associated with viral failure.	2023
Decreasing Time to Antiretroviral Therapy after HIV Diagnosis in a Clinic-Based Observational Cohort Study in Four African Countries	Esber et al.^14^	To Characterize Temporal Trends and Other Factors Influencing the Interval between HIV Diagnosis and ART Initiation	The interval between diagnosis and ART initiation has decreased over time.	2020
Weight Gain During the Dolutegravir Transition in the African Cohort Study	Esber et al.^9^	Evaluated Changes in Weight and BMI after Switch to TLD by PLWH	PLWH on TLD had 1.77 times the hazard of developing a high BMI. The average change in weight prior to the switch was 0.35 kg/year, and in the year after transition to TLD, it was 1.46 kg/year.	2022
Impact of Weight Gain with Dolutegravir on Antiretroviral Adherence and Viral Suppression in Four African Countries	Romo et al.^10^	To Examine the Association between Total Body Weight (TBW) Change after Switching ART to TLD and the Effect on Adherence and Viral Load	TBW change was not significantly associated with nonadherence or becoming virally unsuppressed or detectable.	2023
Socio-Behavioral Factors and Adherence				
Predictors and Barriers to Condom Use in the African Cohort Study	Analogbei et al.^22^	Assessed Condom Use and Barriers to Use	Condom use was more common among older participants, males, PLWH, and those with an HIV-infected partner.	2020
Prevalence and Predictors of Food Insecurity among People Living with and without HIV in the African Cohort Study	Onyenakie et al.^24^	Determined the Prevalence and Identified Predictors of Food Insecurity	Having dependents was associated with food insecurity. Certain sites had more food insecurity than others.	2022
Assessing the Impact of HIV Support Groups on Antiretroviral Therapy Adherence and Viral Suppression in the African Cohort Study	Mbah et al.^20^	Assess the Impact of Support Group Attendance on ART Adherence and Viral Suppressions	There was no association between support group attendance, ART adherence, and viral suppression.	2021
Perceived Satisfaction with HIV Care and Its Association with Adherence to Antiretroviral Therapy and viral suppression in the African Cohort Study	Somi et al.^19^	Examined the Associations between Satisfaction with HIV Care and ART Adherence and Viral Load Suppression	There was no significant difference in viral suppression and satisfaction with care. Satisfaction with HIV care was moderately associated with ART adherence.	2021
Persons Living with HIV in Sero-Discordant Partnerships Experience Improved HIV Care Outcomes Compared with Persons Living with HIV in Sero-Concordant Partnerships: A Cross-Sectional Analysis of Four African Countries	Reed et al.^23^	Assessed the Association between Sero-Discordant Status, ART Uptake, and VL Suppression	PLWH in sero-discordant sexual partnerships demonstrated improved uptake of ART compared with those in sero-concordant partnerships.	2021
Temporal Trends In Self-Reported HIV Stigma and Association with Adherence and Viral Suppression in the African Cohort Study	Esber et al.^18^	Describe Temporal Trends in Self-Reported Experienced Stigma and Association with ART Adherence and Viral Suppression	Stigma was associated with decreased odds of ART adherence and viral suppression.	2022
Routine HIV Clinic Visit Adherence in the African Cohort Study	Dear et al.^21^	Identified Factors Associated with Missed Routine Clinic Visits	Missed clinic visits were associated with alcohol use, a history of incarceration, depression, and viral nonsuppression.	2022
Clinical Similarities and Differences between Two Large HIV Cohorts in the United States and Africa	Monroe et al.^17^	Compared Patient and Clinical Site Characteristics among Adult PLWH Enrolled in Two Longitudinal HIV Cohort Studies—AFRICOS and the DC Cohort	Similar rates of viral suppression, hypertension, hepatitis B co-infection, and alcohol use were observed. AFRICOS participants had significantly higher rates of CD4 <200 and tuberculosis and significantly lower rates of obesity, DM, hepatitis C, co-infection, and syphilis.	2022
Mental Health and Cognition				
Limitations of the International HIV Dementia Scale in the Current Era	Milanini et al.^56^	Define the Performance Characteristics of the International HIV Dementia Scale (IHDS)	The IHDS had poor performance characteristics for the identification of cognitive impairment in East Africa.	2018
Monocyte Activation, HIV, and Cognitive Performance in East Africa	Munoz-Nevarez et al.^46^	Investigated the Association of Cognitive Performance and Immune Activation in PLWH and PLWoH	Higher levels of plasma soluble CD14 (sCD14) were inversely associated with worse cognitive performance in participants in Uganda and Kenya regardless of HIV status. Higher levels of I-FABP were associated with worse cognitive performance in PLWH.	2020
East African HIV Care: Depression and HIV Outcomes	Meffert et al.^28^	Understand the Relationship between Depression and HIV Viral Load	PLWH had a high prevalence of depression. Diverse depression symptoms were independently associated with increases in viral load.	2019
Frequency and Predictors of HIV-Related Cognitive Impairment in East Africa: The African Cohort Study (AFRICOS)	Milanini et al.^30^	Examined Frequency and Predictors of Cognitive Impairment	Literacy, country of residence, WHO stage, and current CD4 cell count were associated with increased risk of cognitive dysfunction.	2020
Non-Communicable Diseases				
Noninfectious Comorbidity in the African Cohort Study	Ake et al.^3^	Understand the Burden of NCDs	PLWH were at high risk for having NCDs with a CD4 count <200.	2019
Ophthalmic Disease Prevalence and Incidence among People Living with Human Immunodeficiency Virus in the AFRICOS Study	Harvey et al.^25^	Examine Prevalence and Incidence of Opthalmic Disease in PLWH	Opthalmic disease was higher in PLWH than in PLWoH. Conjunctivitis was the most common diagnosis.	2021
Non-Communicable Diseases by Age Strata in People Living with and without HIV in Four African Countries	Chang et al.^26^	Examine the Composite Role of Age and HIV Status on NCDs in PLWH and PLWoH	NCDs differed significantly by study site, with participants from Nigeria having the highest odds of elevated BP, dysglycemia, and renal insufficiency.	2022
HIV and Co-Infections				
Factors Associated with Presumptive Sexually Transmitted Infections Among Care-Seeking Adults in the African Cohort Study	Semwogerere, et al.^31^	Describe STI Prevalence and Identify Factors Associated with STIs	Odds of an STI diagnosis were higher among participants 18–29 years old, females, and those with depression. Viral suppression was protective against STIs.	2021
Brief Report: Increased Inflammation and Liver Disease in HIV/HBV-Coinfected Individuals	Copeland et al.^35^	Evaluated the Impact of HIV/HBV Co-Infection on Inflammatory Biomarkers and Liver Disease Severity	MMP-2, MMP-9, and FGF-19 levels were dysregulated among the HIV/HBV–co-infected, and 11% of HIV/HBV–co-infected had evidence of undiagnosed advanced liver disease.	2021
Prevalence and Risk Factors Associated with HIV and Syphilis Co-Infection in the African Cohort Study: A Cross-Sectional Study	Gilbert et al.^32^	To Describe Significant Risk Factors Associated with Serologically Confirmed Syphilis and HIV	Risk factors for HIV and syphilis co-infection included lower educational level and alcohol consumption, whereas ART with preserved VL was protective.	2021
Assessment of Tuberculosis Disease Activity in People Infected with *Mycobacterium tuberculosis* and Living with HIV: A longitudinal Cohort Study	Kroidl et al.^34^	Assessed TB Disease Activity over 5 Years in PLWH Co-Infected with *M. tuberculosis* Using a Surrogate Biomarker.	Activated MTB-specific T cells were present in 63% and 23% of incipient TB cases 6 and 12 months before diagnosis of active diseases. Transient increases of MTB-specific T-cell activation were observed in individuals with latent infections, whereas persistent activations were a hallmark of recurrent TB after the end of treatment.	2022
Genetic Variation That Determines TAPBP Expression Levels Associates with the Course of Malaria in an HLA Allotype-Dependent Manner	Walker-Sperling et al.^36^	Understand the Role of Tapasin Expression in Malaria Pathogenesis	Imputed TAPBP messenger RNA expression levels along with HLA-I allotype-specific tapasin dependence level were associated with malaria outcome. High TAPBP expression was associated with protection among individuals with tapasin-dependent HLA allotypes relative to low tapasin expression.	2022
Epidemiology of Tuberculosis among People Living with HIV in the African Cohort Study from 2013 to 2021	Ganesan et al.^33^	Describe the Prevalence and Incidence of HIV-TB and Factors Associated with Prevalent and Incident TB	Shorter time since HIV diagnosis, being underweight, taking ART <6 months, and having a CD4 count <200 cells/mm^3^ were significantly associated with incident TB. PLWH on TLD were at lower risk of incident TB compared with those on TLE.	2023
Implications of Asymptomatic Malaria Infections on Hematologic Parameters in Adults Living with HIV in Malaria-Endemic Regions with Varying Transmission Intensities	Kamau et al.^37^	Identify Hematologic Abnormalities in Malaria-Asymptomatic PLWH in Regions with Differing Malaria Transmission	Parasite point prevalence significantly differed across study sites. PLWH had higher odds of leucopenia, but this was not attributable to malaria co-infection.	2023
HIV and COVID				
Transient Reductions in Human Immunodeficiency Virus (HIV) Clinic Attendance and Food Security during the Coronavirus Disease 2019 (COVID-19) Pandemic for People Living with HIV in 4 African Countries	Dear et al.^41^	Assessed HIV Care and Food Security in the Context of the COVID-19 Pandemic	In the early COVID-19 period, PLWH were less likely to be adherent to HIV clinic visits and have food insecurity. This relationship did not persist in the late COVID-19 period.	2021
Severe Acute Respiratory Syndrome Coronavirus-2 Antibody Prevalence in People with and without HIV in Rural Western Kenya, January to March 2020	Crowell et al.^38^	Assess the Prevalence of Detectable SARS–CoV-2 Antibodies in PLWH and PLWoH in Kenya	19 (3.3%) individuals had detectable SARS–CoV-2 antibodies but were asymptomatic.	2021
Coronavirus Antibody Responses before COVID-19 Pandemic, Africa and Thailand	Li et al.^39^	To Investigate Coronavirus-Specific Antibody Responses in Participants in AFRICOS and Thailand	1.8% of participants had a detectable IgG response to SARS—CoV-2 spike (S) subunit 2. Among all coronavirus antigens tested, antibody responses were much higher in participants from Africa than in participants from Thailand.	2022
Immunology and HIV Sequencing				
Biomarkers of Inflammation Correlate with Clinical Scoring Indices in Human Immunodeficiency Virus-Infected Kenyans	Letizia et al.^43^	Assess Inflammation between Treated PLWH with Viral Suppression and PLWoH	Compared with HIV-uninfected controls, virologically suppressed HIV-infected participants had higher levels of CCL5, CXCL10, fatty acid binding protein (FABP) 2, fas ligand (FASLG), matrix metalloproteinase (MMP) 1, MMP7, soluble CD14 (sCD14), and soluble CD163 (sCD163) and lower MMP9.	2019
Pretreatment and Acquired Antiretroviral Drug Resistance among Persons Living with HIV in Four African Countries	Crowell et al.^48^	Characterize HIVDR among Antiretroviral Therapy–Naive and Experienced Participants	There was a high burden of resistance to NRTI and NNRTI classes.	2021
Clinical Factors and Outcomes Associated with Immune Non-Response Among Virally Suppressed Adults with HIV from Africa and the United States	Noiman et al.^44^	Understand the clinical Relevance of Immune Non-Response (INR) and Relationship to the Development of Serious Non-AIDS Events in the U.S. Military HIV Natural History Study and AFRICOS	Higher CD4 nadir was associated with decreased odds of INR. INR was associated with an increased risk of serious non-AIDS events.	2022
Dissecting Drivers of Immune Activation in Chronis HIV-1 Infection	Streeck et al.^45^	Evaluated Markers for Immune Activation and Its Distribution in Various Settings	Chronic immune activation in HIV-1 infection was influenced by viral load, sex, age, region, and ART use.	2022
HIV-1 Drug Resistance and Genetic Diversity in a Cohort of People with HIV-1 in Nigeria	Oluniyi et al.^47^	To understand the Genetic Diversity of HIV-1 and Drug Resistance Mutations in Nigeria	CRF02_AG and subtype G were the dominant circulating forms of HIV-1 in Nigeria. No major INSTI mutation associated with dolutegravir resistance was detected.	2022
Women’s Health				
The Pregnancy Factor: The Prevalence of Depression Among Women Living with HIV Enrolled in the African Cohort Study (AFRICOS) by Pregnancy Status	Jones et al.^50^	Describe the Prevalence of Depression among WLWH by Pregnancy Status and Factors Associated with Depression	Visit category (pregnant, postpartum, and nonpregnant) was not independently associated with depression. Depression was lower during pregnancy.	2021
Transitioning Women to First-Line Preferred TLD Regimen Is Lagging in Sub-Saharan Africa	Shah et al.^11^	Examined TLD Transition by Sex across PEPFAR-Supported HIV Care Programs	Transition to TLD was slower in women than in men at certain clinical sites.	2022
Perinatal Depressive Symptoms and Viral Non-Suppression among a Prospective Cohort of Pregnant Women Living with HIV in Nigeria, Kenya, Uganda, and Tanzania	Concepcion et al.^29^	Determine Association between Perinatal Depressive Symptoms and Viral Non-Suppression among WLWH	Depressive symptoms were associated with increased odds of viral nonsuppression.	2023
High-Risk Human Papillomavirus Genotype Distribution among Women Living with and at Risk for HIV in Africa	Chachage et al.^51^	Evaluated HR-HPV Genotypes among Women with and without HIV	Most frequent HR-HPV types among WLWH were HPV-16, HPV-52, and HPV-35. WLWH with CD4 cells <200 cells/*μ*L had increased risk of having HR-HPV.	2023

AFRICOS = The African Cohort study; ART = antiretroviral therapy; BMI = body mass index; BP = blood pressure; DC = District of Columbia; DM = diabetes mellitus; HBV = hepatitis B virus; HLA = Human leukocyte antigens; HIVDR = HIV drug resistance; HR-HPV = high-risk human papillomavirus; INSTI = integrase strand transfer inhibitor; MTB = Mycobacterium tuberculosis; NCD = noncommunicable disease; NRTI = Nucleoside reverse transcriptase inhibitors; NNRTI = non-nucleoside reverse transcriptase inhibitor; PEPFAR = U.S. President’s Emergency Plan for AIDS Relief; PLWH = people living with HIV; PLWoH = people living without HIV; Pub = publication; STI = sexually transmitted infection; TB = tuberculosis; TLE = tenofovir/lamivudine/efavirenz; TAPBP = tapasin; VL = viral load; WLWH = women living with HIV.

In 2019, the combination of tenofovir disoproxil fumarate, lamivudine, and dolutegravir (TLD) was rolled out as the preferred first-line regimen. The AFRICOS was able to monitor and evaluate this transition and its effect on HIV outcomes. We found that participants who switched to TLD had significantly lower rates of viral failure compared with those who did not switch.^8^ However, because of dolutegravir’s association with weight gain, we were concerned that the transition from tenofovir/lamivudine/efavirenz (TLE) to TLD would lead to a decrease in adherence and subsequently viral load suppression. The AFRICOS data showed that a substantial proportion of participants experienced weight gain with a body mass index (BMI) at 1.77 times the rate observed in those on other ART regimens.^9^ Despite this, there was no significant impact on adherence or viral load suppression.^10^ Nevertheless, despite the benefits of TLD, AFRICOS data identified that women were less likely to be switched to this lifesaving regimen than men were.^11^ The importance of these results to PEPFAR clinical programs was substantial; AFRICOS data provided early, robust evidence that switching to TLD was safe and effective, helping to catalyze widespread scale-up. As of 2021, TLD accounted for at least of 80% of antivirals dispensed.^12^

Another key finding relates to predictors of all-cause mortality among PLWH. The causes of death varied, including malignancies, infections, and non–HIV-related conditions. Factors such as a lower CD4 count, increased viral load, recent fever, low BMI, clinical depression, and advanced WHO stage were identified as predictors of mortality. However, each additional year on ART was associated with a reduced hazard ratio for mortality.^4^ Although the findings underscored how mortality rates among PLWH were still three times higher than those among contemporaneous comparators with HIV, they provided compelling evidence from diverse African settings of the impact of ART on all-cause mortality.

Looking at data longitudinally across several indicators, health outcomes have improved since the inception of AFRICOS in 2013.^13^ Consistent with changing guidelines, the interval between diagnosis and ART initiation has decreased over time. Median time to ART initiation decreased from 22.0 months (interquartile range [IQR]: 4.0–77.3 months) among participants diagnosed prior to 2006 to 0.5 month (IQR: 0.2–1.8 months) among those diagnosed in 2016 and later.^14^ Upon more recent analysis, most participants started ART the same day as diagnosis. Consistent with these findings, from 2013 to 2021, the prevalence of CD4 <200 decreased from 10.5% to 3.1%, whereas the percentage on ART increased from 76.6% to 100%.^14^^,^^15^ We also found accompanying decreases in mortality over time with the mortality rate for PLWH reduced from 19.74/1,000 person-years in 2013–2014 to 4.74/1,000 person-years in 2019–2020.^4^

2. *Socio-Behavioral Factors and Adherence:* Socio-behavioral factors impacting HIV treatment adherence include social support systems, food security, satisfaction with care, and the presence of stigma. Understanding how these factors affect adherence is crucial, as they influence the effectiveness of HIV treatment, outcomes, and potentially transmission.

Psychosocial factors affecting individuals within the AFRICOS cohort have been a focal point of research, in alignment with PEPFAR’s strategic commitment to mainstreaming behavioral and social sciences into HIV programming.^16^^,^^17^ One study found that although experiencing stigma was associated with decreased odds of adherence to ART and viral suppression, the prevalence of stigma had declined from 22% at study enrollment in 2013 to below 2% by 2018.^18^ We have not identified an association between clinic satisfaction or support group attendance with adherence to therapy.^19^^,^^20^ However, we did identify that missed visits were more common among those <50 years old, those on ART <2 years, and among those with a history of alcohol use, incarceration, and depression. The most common reasons for missing visits were being busy or transportation concerns.^21^ During the COVID-19 pandemic, PEPFAR rapidly scaled up access to multi‐month dispensing to minimize risk to patients and the burden of having to travel to clinics monthly.

Behavioral factors play a crucial role in reducing the risk of transmission and enhancing adherence to HIV treatment as they foster overall health and minimize complicating factors for disease management. Our study looking at condom use found that 64% of participants reported using a condom during the last sexual encounter with their regular partner. Condom use was influenced by age, sex, HIV status, and HIV status of the partner and was less frequent among those who consumed alcohol.^22^ Regarding disclosure, we found that participants in sero-different relationships were more likely to disclose their status compared with participants in relationships with partners of the same HIV status.^23^

The African Cohort study collects data on environmental, social, and economic factors that influence individual behavior. From these data, we have found that food insecurity was highly prevalent in our cohort though not significantly associated with HIV.^24^ We have also noted differences in outcomes between our rural and urban sites, leading to variations in treatment adherence and overall health management. More analysis is forthcoming to better understand the relationship between environmental factors and health outcomes.

3. *Noncommunicable diseases*: Several papers within the AFRICOS cohort studies have investigated the complex interplay between HIV and noncommunicable diseases (NCDs).^25^ This emerging area of research recognizes that PLWH also face an increased risk of developing NCDs such as cardiovascular diseases, diabetes, and certain cancers.

Our analysis found that NCDs were geographically variable and largely consistent with metabolic complications of first-line ARTs.^3^ Building on this analysis and examining the role of age and NCDs, we found that compared with people without HIV aged <50 years, PLWH aged ≥50 years had increased adjusted odds of having diabetes, dysglycemia, and renal insufficiency.^26^ We have also commenced analyses to understand how NCDs are managed and to understand potential unmet non–HIV-related health needs. From 2013 through 2023, approximately 20% of PLWH met our definition of hypertension, of whom about 60% did not receive any blood pressure–lowering medication.^27^ This treatment gap was similar when comparing people with and without HIV and varied by site, likely reflecting differences in how ART clinics manage non–HIV-related conditions. We also found that among those receiving treatment, blood pressure control was suboptimal.

4. *Mental Health and Cognition:* Cognitive impairments can affect an individual’s ability to adhere to complex medication regimens, impacting overall health outcomes. In addition, the psychological burden of living with HIV, including stigma and potential social isolation, can affect mental health, necessitating integrated care approaches that address both psychological and physical aspects of the disease.

The AFRICOS data revealed that depression is a common comorbidity among PLWH, with prevalence rates varying across regions and demographics. Decreasing age, female sex, worse ART adherence, a detectable viral load, and all-cause mortality were all associated with depression.^4^^,^^28^ Among pregnant women, 21% reported depressive symptoms, which were associated with two times greater odds of viral nonsuppression.^29^ The AFRICOS data also shed light on how ART adherence is influenced by mental health, underlining the importance of psychological support in HIV treatment programs.

As PLWH are living longer, understanding the role of HIV’s effect on brain function, leading to challenges in memory, attention, problem-solving abilities, and cognition is important. The AFRICOS data found that 38% of PLWH were cognitively impaired compared with 26% of PLWoH. Factors associated with an increased risk of cognitive impairment included literacy, country, WHO stage 4, and current CD4 cell count.^30^

5. *HIV and Co-Infections*: Co-infections remain a significant challenge for PLWH, and the AFRICOS cohort has been instrumental in unraveling the dynamics of co-infections. Papers within this realm have focused on the prevalence of co-infections, such as tuberculosis (TB), hepatitis, and sexually transmitted infections (STIs), and their impact on HIV progression and treatment outcomes.

The AFRICOS cohort had an STI prevalence of 7.7% which did not differ by HIV status. However, the odds of having an STI were higher among those with a lower education level, females, younger individuals, and those with depression.^31^ Risk factors associated with co-infection with syphilis specifically included lower education levels and alcohol consumption.^32^

With respect to TB, between 2013 and 2021, the TB prevalence was 3% and the incidence rate was 600 per 100,000 person-years. Factors significantly associated with prevalent TB included higher educational attainment, being underweight, and having a CD4 count <200 cells/mm^3^.^33^ Notably, individuals on TLD had a 78% lower risk of incident TB compared with those on TLE. To better understand the dynamics of subclinical disease, we looked at longitudinal samples of blood mononuclear cells from individuals with active and recurrent TB, latent infection, or incipient TB. The data revealed that Mycobacterium tuberculosis-specific CD4 T-cell activation can distinguish active TB from latent TB with a sensitivity and specificity of 86%. This activation decreases upon TB treatment initiation. Progression to active TB disease began 6–12 months before clinical diagnosis, suggesting that blood biomarkers could aid in early TB detection.^34^

Co-infection with hepatitis and HIV complicates the clinical management of patients and requires careful coordination of treatment strategies to minimize liver damage. The AFRICOS data found that individuals co-infected with HIV and hepatitis B virus (HBV) exhibited higher levels of inflammation than those with either infection alone or uninfected individuals. This inflammation was linked to liver disease, with notable dysregulation in biomarkers matrix metalloproteinase-2, matrix metalloproteinase-9, and fibroblast growth factor-19. In addition, the co-infected group had a higher Fibrosis-4 score, suggesting undiagnosed advanced liver disease, indicating a more complex and severe disease progression in HIV/HBV co-infection.^35^

Our work on malaria found that two single-nucleotide polymorphisms (SNPs) significantly influence tapasin Messenger RNA expression levels in Africans. These SNPs are associated with a lower prevalence and incidence of clinical malaria, particularly in individuals carrying tapasin-dependent human leukocyte antigen (HLA) class I molecules allotypes. This suggests that enhanced antigen presentation, influenced by genetic variations, can affect the course of malaria, providing important insights for vaccine efficacy evaluation.^36^^,^^37^

6. *HIV and COVID*: The emergence of the COVID-19 pandemic presented an unprecedented challenge to global health systems, including those already addressing the complexities of HIV. The AFRICOS cohort was able to illustrate the intricate relationship between COVID-19 and HIV, shedding light on how PLWH were affected by and responded to the pandemic.

Disruptions in healthcare services due to lockdowns and resource reallocations to the COVID-19 response affected HIV testing, treatment initiation, and adherence to ART. African Cohort Study activities were paused on March 19, 2020 to establish safety procedures for streamlined telephonic and subsequently in-person visits. The first telephonic visit was on May 7, 2020 with reopening dates varied by program. To document the effects of the pandemic on HIV, the AFRICOS protocol was amended to capture data related to COVID, including vaccination status, knowledge, attitude, and beliefs regarding vaccination and the ability to do nasal swab testing.

Initially, there was widespread concern that COVID-19 would have devastating effects in Africa. However, some countries in Africa demonstrated surprising resilience. There are many hypotheses regarding why many countries in Africa were able to evade widespread dissemination. Because AFRICOS participants provided samples for storage and retrospective testing, we were able to analyze retrospective samples to better understand circulating antibodies and assess infections that may have gone undetected. The prevalence of SARS–CoV-2 antibodies in western Kenya among stored samples was 3.3%. When matched with self-reported symptoms, only one person was symptomatic, suggesting asymptomatic infections may have been circulating prior to the first reported case.^38^ When we compared AFRICOS samples with those from our Thailand studies, we found coronavirus antibodies were much higher among AFRICOS participants than among those from Thailand, especially for outbreak coronaviruses.^39^ By using retrospective and prospective samples, we are able to better understand how COVD-19 affected diverse communities and the effectiveness of public health interventions and to track mutation patterns that can inform vaccine and treatment development. As such, AFRICOS offers a compelling model for how investment in health research infrastructure can be effectively and rapidly leveraged to advance a broader global health security agenda.

There was also concern that COVID-19’s disruption to public health services would affect HIV care and treatment outcomes.^40^ The AFRICOS data revealed that clinic attendance, food security, and viral load suppression decreased early in the pandemic when control measures were most stringent.^41^^,^^42^ As the pandemic progressed, PEPFAR implemented several changes to providing care and treatment, including community medication dispensaries, outdoor waiting rooms, and allowance for multiple months of medication to be dispensed at a time. As the pandemic progressed, data showed that adherence was unaffected, and food security stabilized and viral load increased later in the pandemic.^41^^,^^42^

7. *Immunology and HIV Sequencing:* These studies aimed to decipher the role of host-virus interactions, including the characteristics of viruses and immune responses that influence HIV acquisition, progression, and clinical outcomes.

The African Cohort study has characterized associations between inflammatory markers and disease outcomes. For example, we found that virally suppressed PLWH had higher levels of nonspecific inflammatory markers (CCL5, CXCL10, fatty acid binding protein (FABP), fas ligand (FASLG), matrix metalloproteinase (MMP) 1, MMP7, soluble (s)CD14, and sCD163) compared with PLWoH. Some of these biomarkers correlated with clinical scoring indices predictive of morbidity and mortality, such as the Veterans Aging Cohort Study index.^43^ Inflammatory markers were even higher among viremic participants, with the magnitude and character of immune activation influenced by factors such as viral load, sex, age, CD4 count, and region.^44^ Specific comorbid conditions—such as viral hepatitis, syphilis, and TB—contributed to phenotypically unique patterns of chronic inflammation.^45^ Tuberculosis-specific T-cell activation also correlated with disease activity. The AFRICOS data suggest that CD38 expression on TB-specific CD4 T-cells may be a useful surrogate biomarker to detect incipient TB and predict TB recurrence after treatment.

We also looked at biomarkers to characterize HIV-related cognitive outcomes to help guide early detection and prevention efforts. We identified that sCD14 was inversely correlated to neuropsychological testing performance (NTP) for both PLWH and PLWoH. In PLWH, I-FABP was inversely associated with NTP, regardless of viral suppression. These results confirm that inflammatory sources contribute to cognitive performance regardless of HIV status.^46^ More work is needed to determine how these biomarkers can be longitudinally used to guide targeted treatment and interventions.

The African Cohort study works with collaborators to understand the genetic diversity of HIV as it influences the virus’s transmissibility and its response to treatment, thereby informing the development of effective therapy and vaccine strategies. Among Kenya and Uganda AFRICOS participants, HIV-1 subtype A is the main circulating virus, with subtype D contributing a minority. In Tanzania, the majority are subtype C, with a minority being subtype A. In Nigeria, subtypes G and CRFC02_AG are the predominant circulating subtypes.^47^ From 2013 to 2019, AFRICOS identified an almost 3-fold increase in pretreatment drug resistance in Uganda, from 8.0% to 24.3%, driven by increases in resistance to both Nucleoside reverse transcriptase inhibitors and non-nucleoside reverse transcriptase inhibitors. African Cohort Study data also showed that the vast majority of viremic ART-experienced participants had drug-resistant viruses that potentially limited the efficacy of standard first- and second-line regimens. These findings informed country-specific guidelines on standardized ART regimens and the urgency to consider switching from failing regimens.^48^

More recently our preliminary drug resistance testing among individuals on TLD for more than 6 months who had viral nonsuppression (*N* = 133/2,004) and were successfully virally amplified (*N* = 65/133) found that 32 (49%) had a drug mutation. There was an integrase inhibitor mutation in seven (11%) specimens, two (3%) with a mutation-conferring high-level dolutegravir resistance.^49^

8. *Women’s Health:* Women face unique needs and challenges, including access to reproductive health services, mental health interventions, and tailored services perinatally.

Perinatal depression can affect a mother’s ability to adhere to HIV treatment regimens, potentially leading to viral nonsuppression and increased risk of vertical transmission of HIV. However, there are few data regarding antenatal, prenatal, and perinatal trajectories of depression and viral suppression. We found that throughout the 1-year preconception phase, 86% had elevated depressive symptoms, 92% during pregnancy and 94%postpartum.^50^ In a multivariate analysis, elevated depressive symptoms were associated with viral nonsuppression.^29^

With respect to HPV, we found the prevalence of HPV was 50.9% among women living with HIV (WLWH) compared with 38.1% in women living without HIV (WLWoH) in the AFRICOS cohort. The most common high-risk human papillomavirus (HR-HPV) types among WLWH were HPV-16 (13.5%), HPV-52 (9.5%), and HPV-35 (9.3%). The study also found that HR-HPV infection was more frequent among Tanzanian WLWH and among women with a CD4^+^ T cell count <200 cell/*μ*L.^51^

## LOOKING TO THE FUTURE

### Sustaining the gains and accelerating the response.

The global HIV response is at a pivotal moment. Tremendous progress informed by science, including AFRICOS, has allowed more people to initiate HIV treatment than ever before. Nonetheless, gaps in HIV treatment services remain in specific countries and populations. As countries progress toward the United Nations’ 95-95-95 targets, there are critical clinical, biobehavioral, and epidemiologic knowledge gaps that must be closed and where AFRICOS can continue to shine a light that guides policy decisions. The PEPFAR’s 5-year strategic plan outlines a commitment to ensuring that its programs continue to “follow the science,” including leveraging the AFRICOS platform to address emerging challenges.^16^

#### Adolescents and youth.

Demographic trends across the continent indicate a pressing need to understand how to optimize clinical services to adolescents and young adults, especially those with perinatally acquired HIV who are at the highest risk of treatment interruption and treatment complications and who face psychosocial barriers to care.^52^^,^^53^ In 2020, AFRICOS expanded eligibility into the cohort to include individuals 15 years and older (previously restricted to 18 years and older). Ongoing analyses seek to focus on the younger cohort and understand the role of food security, stigma, mental health, and cognitive development on HIV outcomes. The AFRICOS’s increasing focus on behavioral science, including understanding how to improve and sustain changes in behavior, norms, and the enabling environment, will be especially relevant to this vulnerable population.

#### Drug-resistant HIV.

Despite the incredible success of TLD, AFRICOS along with other studies have indicated that treatment interruptions and drug resistance are emerging clinical phenomenon with varying prevalence of resistance by country.^47^^,^^48^^,^^54^^,^^55^ Although our preliminary data presented above found limited high-level resistance to dolutegravir resistance, AFRICOS has prioritized research to understand the determinants of drug resistance as well as optimize analytic tools for predicting those at greatest risk for treatment interruption.^49^ We are also working with partners to understand how other diagnostic tools, such as urine drug testing or adherence counseling, can help identify individuals who may be at risk for drug resistance.

#### Increasing prevalence of NCDs.

Ongoing research is looking at the role of inflammation among individuals with any detectable viral load in the development of NCDs. Future NCD analysis will delve into the relationship between HIV and cardiovascular disease, aiming to understand the long-term impacts of HIV on heart health and develop targeted prevention and treatment strategies. In addition, we hope to better understand how mental health and diabetes are being addressed and treated in HIV clinics.

#### Cognition and HIV.

As individuals live longer with HIV, AFRICOS is examining the intersection of cognition, aging, and HIV. This intersection demands a more nuanced approach to treatment and care, focusing on early detection of cognitive impairment and tailored strategies to manage the combined effects of HIV and aging on brain health. Unfortunately, we found that the International Dementia Scale has a high false-positive rate for the identification of cognitive impairment in East Africa.^56^ We plan to reevaluate our screening tools and make protocol adjustments to better capture changes in cognition and mobility.

## OTHER CONTRIBUTIONS AND CAPACITY BUILDING

Many AFRICOS analyses have been led by site investigators. In addition to building scientific capacity and promoting career growth, AFRICOS has supported sites with developing new technical and laboratory capabilities, including HIV sequencing and drug resistance testing, HPV sample collection, microbiome sample collections (stool, rectal/vaginal swabs), and Mpox sample collection. In alignment with PEPFAR’s commitment to transition toward sustainability, AFRICOS envisions a plan for increasing leadership of its science priorities, data analysis, and presentation of accomplishments by scientific leaders across the four partner countries.

## CONCLUSION

In summary, the body of work emanating from the AFRICOS cohort has contributed to the global knowledge base, informing evidence-based interventions, policy decisions, and further research directions in the ongoing efforts to combat HIV and improve the lives of PLWH in the region. Moreover, AFRICOS serves as one of the best examples of PEPFAR’s commitment to investing in science and data to drive programming decisions. The AFRICOS findings have had a substantial impact on PEPFAR program priorities over the last decade. Looking to the future, the network is ideally placed to inform broader global health strategies in the ongoing fight against HIV. As the global community strives toward the UNAIDS 95-95-95 goals, the nuanced insights from cohort studies can be instrumental in refining and optimizing global strategies. The AFRICOS cohort serves as a catalyst for ongoing dialogue and collaboration, encouraging the exchange of best practices, innovative approaches, and culturally relevant interventions.
